# Let’s See Day Three: Association and Potential Discriminatory Value of Selected Inflammatory and Immunonutritional Biomarkers for Postoperative Morbidity After Minimally Invasive Colorectal Cancer Surgery

**DOI:** 10.3390/nu18152581

**Published:** 2026-08-06

**Authors:** Magdalena Leśniewska, Zofia Chyła, Zuzanna Dąbrowska, Jolanta Sado, Kinga Bielarska, Szymon Bielecki, Zuzanna Pelc, Marcin Kubiak, Katarzyna Sędłak, Radosław Mlak, Karol Rawicz-Pruszyński

**Affiliations:** 1 Doctoral School, Medical University of Lublin, Chodźki 7 St., 20-093 Lublin, Poland; d.magdalenalesniewska@umlub.edu.pl; 2 Department of Surgical Oncology, Medical University of Lublin, Staszica 16 St., 20-080 Lublin, Poland; 61623@umlub.edu.pl (Z.C.); 61934@umlub.edu.pl (Z.D.); 63286@umlub.edu.pl (J.S.); 61770@umlub.edu.pl (K.B.); 63151@umlub.edu.pl (S.B.); zuzanna.pelc@umlub.edu.pl (Z.P.); marcin.kubiak@umlub.edu.pl (M.K.); katarzyna.sedlak@umlub.edu.pl (K.S.); 3 Department of Laboratory Diagnostics, Medical University of Lublin, Chodźki 1 St., 20-093 Lublin, Poland; radoslaw.mlak@umlub.edu.pl

**Keywords:** colorectal cancer, elderly patients, postoperative complications, neutrophil-to-lymphocyte ratio, HALP score, Host Index, inflammation, immunonutrition, frailty, Comprehensive Complication Index

## Abstract

**Background:** Composite immunonutritional biomarkers reflecting systemic inflammation, nutritional status, and immune competence have emerged as promising tools for perioperative risk stratification in colorectal cancer (CRC). However, their prognostic value during the perioperative period following minimally invasive CRC surgery remains unresolved. This study aimed to evaluate the association between perioperative inflammatory biomarkers, including the neutrophil-to-lymphocyte ratio (NLR), hemoglobin–albumin–lymphocyte–platelet (HALP) score, and Host Index (H-index), and short-term postoperative outcomes after minimally invasive colorectal cancer surgery. **Methods:** This retrospective single-center study included 105 patients who underwent minimally invasive curative-intent surgery for CRC between 2023 and 2025. Biomarkers were assessed preoperatively and during the first four postoperative days. Severe postoperative morbidity was defined as a Comprehensive Complication Index (CCI) ≥ 20.9. **Results:** Among all parameters, NLR measured on postoperative day 3 demonstrated the highest apparent discriminatory ability for severe complications (AUC = 0.87, 95% CI: 0.74–0.95; sensitivity 91.7%; specificity 73.7%). Preoperative HALP was the most informative preoperative marker (AUC = 0.78, 95% CI: 0.66–0.87; sensitivity 76.5%; specificity 87.7%). **Conclusions:** Perioperative inflammatory and immunonutritional biomarkers are associated with postoperative morbidity following CRC surgery. Preoperative HALP and postoperative day 3 NLR demonstrated the strongest apparent discrimination for CCI ≥ 20.9; however, these findings require confirmation in larger, independent prospective cohorts.

## 1. Introduction

Systemic inflammation, malnutrition, anemia, and impaired immune function are clinically correlated and substantially influence postoperative recovery and long-term oncological outcomes in colorectal cancer (CRC). Consequently, composite immunonutritional biomarkers derived from routine laboratory tests have emerged as practical and inexpensive tools for perioperative risk stratification. Among these, the neutrophil-to-lymphocyte ratio (NLR) and the hemoglobin–albumin–lymphocyte–platelet (HALP) score reflect the complex interaction between inflammatory status, nutritional reserve, and host immune competence [1,2,3,4,5,6,7].

Several studies have demonstrated the prognostic value of these markers in CRC. Biologically, an elevated NLR reflects a systemic inflammatory state characterized by neutrophilia and relative lymphopenia, indicating enhanced innate inflammatory activity together with impaired adaptive antitumor immunity. Surgical trauma further amplifies this response through cytokine release and neuroendocrine stress, potentially increasing tissue injury, susceptibility to infection, and impaired postoperative recovery. Conversely, low HALP, GNRI, and PNI values reflect the combined effects of malnutrition, anemia, and immune dysfunction, suggesting reduced physiological reserve to withstand both cancer-related inflammation and the metabolic demands of surgery. Josse et al. reported that a preoperative NLR ≥ 2.3 independently predicted major postoperative complications following colorectal surgery. Similarly, Malietzis et al. found that elevated preoperative NLR was associated with poorer disease-free survival after curative CRC resection. Furthermore, a recent meta-analysis by Xu et al. including more than 13,000 patients confirmed that low HALP scores are consistently associated with inferior survival across multiple solid malignancies, supporting the clinical relevance of combined immunonutritional indices. Recent evidence also suggests that nutritional indices such as GNRI and PNI predict postoperative complications and survival in elderly patients undergoing colorectal cancer surgery [7,8].

Accurate perioperative risk stratification remains essential in colorectal cancer surgery because postoperative outcomes are influenced by a complex interplay of systemic inflammation, nutritional status, physiological reserve, and patient-related factors. Although chronological age is traditionally considered an important determinant of surgical risk, biological reserve and frailty are more informative predictors of postoperative outcomes. Comprehensive Geriatric Assessment (CGA) remains the recommended standard for evaluating biological age; however, its routine implementation is limited by time and resource requirements. Consequently, simple laboratory-derived immunonutritional biomarkers may provide an objective and readily available adjunct for perioperative risk assessment, complementing rather than replacing comprehensive clinical evaluation [9,10,11,12,13,14,15,16].

Although HALP, NLR, and H-index have been investigated in patients with colorectal cancer, previous studies have commonly evaluated individual biomarkers, focused primarily on preoperative measurements, or used heterogeneous definitions of postoperative morbidity. Moreover, most available evidence is derived from Asian cohorts, while data from European populations remain limited. Direct comparisons of their perioperative trajectories and associations with comprehensive postoperative morbidity after minimally invasive colorectal cancer surgery remain scarce.

Therefore, this study aimed to evaluate the associations between perioperative HALP, NLR, and H-index values and postoperative morbidity following minimally invasive colorectal cancer surgery, with additional assessment of their diagnostic discrimination and performance in older patients. We hypothesized that unfavorable perioperative biomarker profiles would be associated with a higher risk of severe postoperative morbidity.

## 2. Materials and Methods

### 2.1. Study Design

This retrospective, single-center study was conducted at a tertiary university hospital in Eastern Poland. Approximately 150 patients with colorectal cancer (CRC) undergo surgical treatment annually at our institution, defining it as a high-volume referral center [17]. Data were obtained from a prospectively maintained institutional database covering the period from January 2023 to May 2025. The study was approved by the Institutional Review Board (KE-0254/331/2018) and conducted in accordance with the Declaration of Helsinki.

### 2.2. Participants

**Figure 1:**
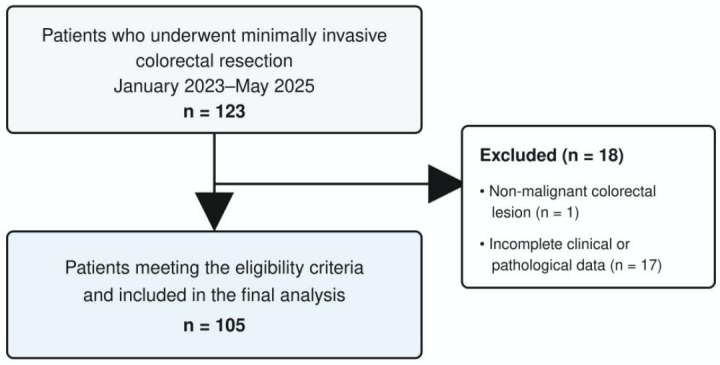
Flowchart of the study.

Consecutive adult patients with histologically confirmed CRC, clinically staged as cT2–4N0–2M0, who underwent minimally invasive curative-intent resection between January 2023 and May 2025 were included in the study. The inclusion criteria were age ≥ 18 years and histopathologically confirmed colorectal cancer. Adult patients were eligible if they had histologically or clinically confirmed colorectal cancer staged as cT2–4N0–2M0 and underwent elective laparoscopic or robot-assisted resection with curative intent. Patients were excluded in cases of benign disease, distant metastases, or insufficient clinical or laboratory data required for the principal analyses (Figure 1).

### 2.3. Outcomes and Definitions

The primary outcome of our study was the occurrence of clinically relevant postoperative morbidity defined as CCI ≥ 20.9. This threshold corresponds to the morbidity burden of at least one Clavien–Dindo grade II complication and has been used in previous surgical outcome studies as a clinically meaningful cutoff for postoperative morbidity [18]. The secondary outcome of the study was Length of Stay (LOS) [19].

### 2.4. Data Collection and Biomarker Assessment

Data were collected on patient demographics, body mass index (BMI), comorbidities, surgical approach, intraoperative leak test, intraoperative complications, postoperative complications, anastomotic leakage, ICU stay, LOS, CCI, tumor grade, clinical and pathological TNM stage, tumor regression grade, resection margin status, number of harvested and metastatic lymph nodes, operative time, neoadjuvant treatment, and adjuvant chemotherapy. Laboratory parameters were collected preoperatively and on postoperative days 1, 2, 3, and 4, when available. The analyzed parameters included C-reactive protein (CRP), albumin, procalcitonin, hemoglobin, red blood cell count, neutrophil count, lymphocyte count, monocyte count, white blood cell count, platelet count, NLR, HALP, and Host Index.

The neutrophil-to-lymphocyte ratio (NLR) was calculated as the absolute neutrophil count divided by the absolute lymphocyte count [20].

HALP was calculated using hemoglobin, albumin, lymphocyte count, and platelet count according to the following formula: HALP = (hemoglobin × albumin × lymphocyte count)/platelet count [21].

The Host Index was calculated using hemoglobin, albumin, lymphocyte count, and platelet count according to the following formula: H-index = (neutrophil count × monocyte count)/(Hb × albumin × lymphocyte count) × 100 [22].

### 2.5. Age-Stratified Analysis

For the age-stratified analysis, patients were divided into two groups according to age at surgery: ≥65 years and <65 years. The association between inflammatory and immunonutritional biomarkers and short-term postoperative outcomes was assessed separately in both age groups to determine whether biomarker performance differed according to chronological age.

### 2.6. Statistical Analysis

Statistical analysis was performed using Statistica version 13 (TIBCO Software Inc., Palo Alto, CA, USA) and MedCalc version 22.021 (MedCalc Software Ltd., Ostend, Belgium). Continuous variables were assessed for normality using the Shapiro–Wilk test. As most variables did not follow a normal distribution, continuous data are presented as median and interquartile range (IQR) and compared using the Mann–Whitney U test. Correlation between continuous variables was performed using Spearman’s rank correlation test. Categorical variables are expressed as numbers and percentages (%) and compared using the Pearson χ^2^ test or Fisher’s exact test, as appropriate. Patients were divided into two groups according to the severity of postoperative morbidity: CCI < 20.9 and CCI ≥ 20.9. Clinical, pathological, operative, and laboratory variables were compared between groups. The predictive performance of inflammatory and immunonutritional markers, including the H-index, hemoglobin–albumin–lymphocyte–platelet (HALP) score, and neutrophil-to-lymphocyte ratio (NLR), was evaluated using receiver operating characteristic (ROC) curve analysis. The discriminatory ability of each parameter was assessed by calculating the area under the ROC curve (AUC) with corresponding 95% confidence intervals (95% CI). Optimal cutoff values were determined using the Youden index, and sensitivity and specificity were calculated for each threshold. All statistical tests were two-sided, and a *p*-value < 0.05 was considered statistically significant.

## 3. Results

### 3.1. Clinical and Demographic Characteristics of the Study Group

A total of 105 patients undergoing surgery for colorectal cancer were included in the final analysis. Men accounted for 51.4% of the cohort, and the median age was 65 years, with 53.3% of patients aged ≥65 years. The median BMI was 26.2 kg/m^2^. Most patients had comorbidities (84.1%) and underwent laparoscopic surgery (61.0%), whereas robotic surgery was performed in 39.0%. Postoperative complications occurred in 16.2% of patients, anastomotic leakage in 6.7%, and ICU stay was required in 2.9%. The median CCI was 8.7, and the median LOS was 5 days. R0 resection was achieved in 97.1% of patients. Baseline demographic characteristics, including sex, BMI, comorbidities, surgical approach, operative time, histological grade, and neoadjuvant treatment, did not differ significantly between groups (all *p* > 0.05). Similarly, the distribution of surgical procedures, including right hemicolectomy, left hemicolectomy, sigmoidectomy, anterior resection, and ASAR, was comparable between groups (*p* = 0.677). Patients with cT4 tumors were more likely to develop severe postoperative morbidity than patients with lower clinical T stages (46.2%; *p* = 0.0359). Characteristics are presented in Table 1.

### 3.2. Comparison of Clinical Variables and Laboratory Parameters Between Patients with CCI < 20.9 and ≥20.9 in the Early Postoperative Period

Patients with severe postoperative morbidity (CCI ≥ 20.9) had higher rates of ICU admission (12.5% vs. 0%; *p* = 0.0013) and anastomotic leakage (29.2% vs. 0%; *p* < 0.0001) than those with CCI < 20.9. They also had a longer median hospital stay (6 vs. 5 days; *p* = 0.0006). Patient characteristics according to postoperative morbidity are summarized in Table 1.

Preoperatively, patients with CCI ≥ 20.9 had higher CRP concentrations, H-index values, and NLR values, as well as lower hemoglobin levels, lymphocyte counts, and HALP values (all *p* < 0.05). Significant between-group differences in inflammatory, hematological, and immunonutritional parameters were also observed during the first three postoperative days. Notably, the severe morbidity group had lower HALP values on postoperative day (POD) 1 (0.1 vs. 0.2; *p* = 0.0077) and higher H-index (4.5 vs. 1.9; *p* = 0.0056) and NLR values (9.7 vs. 3.4; *p* = 0.0001) on POD3. By POD4, only CRP concentrations and hemoglobin levels remained significantly different between the groups. Detailed laboratory findings are presented in Table 2.

### 3.3. Diagnostic Performance of Inflammatory and Immunonutritional Biomarkers for Predicting Severe Postoperative Morbidity

**Figure 2:**
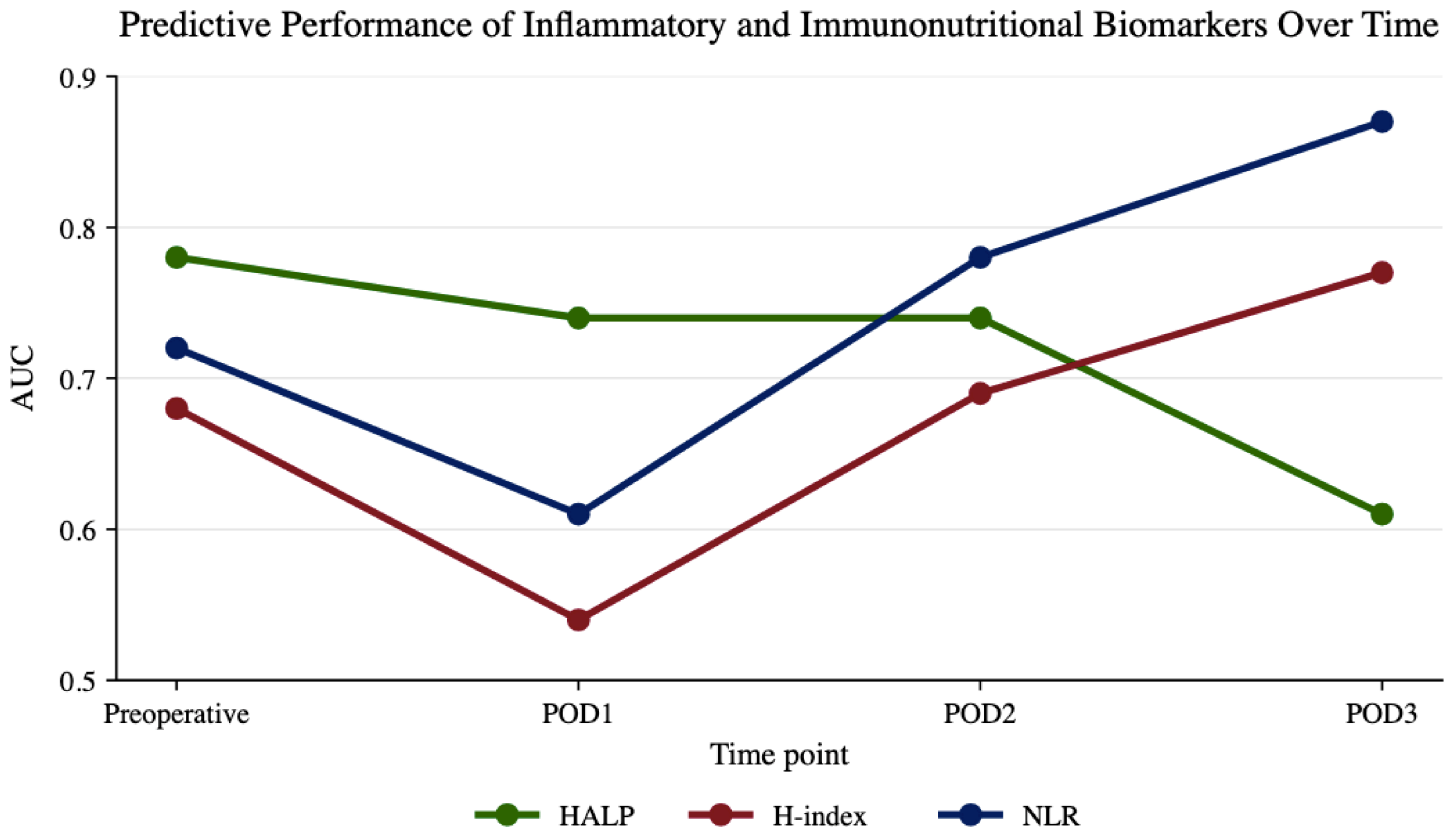
Performance of inflammatory and immunonutritional biomarkers for predicting severe postoperative morbidity.

In the analysis of the diagnostic utility of inflammatory markers for predicting severe postoperative complications, NLR measured on postoperative day 3 demonstrated the highest AUC among all analyzed parameters (AUC = 0.87; 95% CI: 0.74–0.95; *p* < 0.0001), with a sensitivity of 91.67% and specificity of 73.68% at a cutoff value > 5.23 (Figure 2).

Among preoperative markers, HALP demonstrated the highest diagnostic value (AUC = 0.78; 95% CI: 0.66–0.87; *p* = 0.0003), while also exhibiting the highest specificity among preoperative parameters (87.7%) and a sensitivity of 76.5% at a cutoff value ≤ 0.19. Preoperative NLR showed lower sensitivity (55%) but high specificity (82.76%), with an AUC of 0.72 (95% CI: 0.60–0.81; *p* = 0.0022). In contrast, preoperative H-index was characterized by high sensitivity (80%) but relatively low specificity (51.72%), resulting in only moderate diagnostic performance (AUC = 0.68; 95% CI: 0.57–0.78; *p* = 0.0081).

On postoperative day 1, HALP demonstrated the best diagnostic performance among the analyzed parameters (AUC = 0.74; 95% CI: 0.60–0.85; *p* = 0.0074), achieving a sensitivity of 73.3% and a high specificity of 86.11% at a cutoff value ≤ 0.15. On postoperative day 2, HALP demonstrated the highest sensitivity (86.67%), whereas NLR achieved the highest AUC (AUC = 0.78; 95% CI: 0.63–0.89; *p* = 0.0001), with a sensitivity of 88.24% and specificity of 62.96% at a cutoff value > 5.70.

The highest sensitivity among all analyzed parameters was observed for H-index on postoperative day 3 (100%); however, its low specificity (47.37%) limited its clinical utility despite statistically significant discriminatory performance (AUC = 0.77; 95% CI: 0.63–0.88; *p* = 0.0003). Overall, HALP demonstrated the highest specificity, whereas NLR presented the strongest association with the occurrence of severe postoperative complications. Characteristics are presented in Table 3.

### 3.4. Age-Dependent Predictive Value of HALP, NLR, and H-Index in the Achievement of TO/TOO

In the study cohort, there was no evidence that HALP, NLR, or H-index demonstrated clearly greater prognostic relevance in the elderly population, defined as patients aged ≥65 years, with respect to the achievement of TO/TOO. In the subgroup analysis of patients aged ≥65 years, H-index, HALP, and NLR values did not differ significantly between patients who achieved TO and those who did not (H-index: *p* = 0.8674; HALP: *p* = 0.4124; NLR: *p* = 0.4360). Similarly, no significant differences were observed for TOO within the same age group (H-index: *p* = 0.8137; HALP: *p* = 0.4972; NLR: *p* = 0.3458).

## 4. Discussion

The present study demonstrated that inflammatory and immunonutritional biomarkers, particularly HALP and NLR, are significantly associated with the severity of postoperative complications after colorectal cancer (CRC) surgery. Among the analyzed parameters, postoperative NLR and preoperative HALP showed the highest predictive performance for severe postoperative morbidity defined as CCI ≥ 20.9.

Our findings support the growing evidence that systemic inflammation is strongly associated with postoperative morbidity after CRC surgery [3,6,23]. Patients with severe postoperative complications demonstrated significantly higher preoperative CRP, NLR, and H-index values, together with lower HALP values, suggesting that baseline inflammatory and nutritional status may substantially influence perioperative recovery. Similar observations have been reported in multiple studies evaluating inflammatory indices in colorectal surgery. A study by Cook et al. demonstrated that postoperative NLR ≥ 9.3 on postoperative day 1 was associated with significantly increased complication risk following elective colorectal surgery [24]. Likewise, subsequent studies confirmed that elevated perioperative NLR reflects excessive inflammatory response and impaired immune regulation, both associated with infectious complications and prolonged recovery [25]. In the present cohort, postoperative day 3 NLR demonstrated the highest predictive value among all analyzed biomarkers (AUC = 0.87), representing the strongest single predictor of severe postoperative complications. These findings are consistent with contemporary evidence showing that postoperative inflammatory dynamics may outperform preoperative measurements in predicting adverse outcomes. Ortíz-López et al. recently demonstrated that persistently elevated postoperative NLR after CRC surgery was independently associated with worse prognosis and recurrence risk, highlighting the biological importance of sustained postoperative systemic inflammation [26]. Similarly, previous reviews suggested that both preoperative and postoperative NLR are associated with infectious complications, anastomotic leakage, and prolonged hospitalization after colorectal surgery [27].

An important finding of our study was the strong prognostic utility of HALP, particularly in the preoperative setting. HALP combines hemoglobin, albumin, lymphocyte count, and platelet count, thereby reflecting nutritional reserve, systemic inflammation, anemia, and immune competence simultaneously. In our cohort, preoperative HALP achieved the highest specificity among all preoperative markers and remained significantly associated with severe postoperative morbidity. These observations are consistent with recent reports emphasizing the prognostic role of nutritional–inflammatory indices in CRC. Similar findings have been demonstrated for related scores such as the Prognostic Nutritional Index (PNI) and the Naples Prognostic Score (NPS). Kor et al. showed that lower preoperative PNI independently predicted postoperative complications, anastomotic leakage, and poorer overall survival in CRC patients undergoing curative surgery [8]. Likewise, Park et al. demonstrated in Cancers that postoperative NPS provided higher prognostic accuracy than preoperative inflammatory markers in stage II-III CRC patients [28]. These findings support the concept that combined nutritional–inflammatory scores may better reflect physiological reserve than isolated inflammatory markers alone.

Interestingly, although elderly patients are traditionally considered more vulnerable to postoperative morbidity, our study did not confirm substantially greater prognostic performance of HALP, NLR, or H-index specifically in patients aged ≥65 years. Only partial associations with complication burden assessed using CCI were observed in this subgroup. This finding may suggest that frailty and biological age are more clinically relevant than chronological age itself. Similar conclusions have been emphasized in contemporary geriatric oncology literature, where frailty assessment and comprehensive geriatric evaluation are increasingly recommended before CRC surgery [29].

Another clinically relevant observation was that inflammatory biomarkers were more strongly associated with severe postoperative complications than with textbook outcome (TO/TOO). This likely reflects the multifactorial nature of TO metrics, which include not only biological complications but also organizational and perioperative management factors. Therefore, inflammatory markers may primarily reflect physiological response to surgical stress rather than global quality-of-care indicators [30].

The analysis of anastomotic leakage demonstrated that postoperative rather than preoperative inflammatory parameters had the greatest predictive value. Patients with leakage exhibited markedly elevated postoperative CRP and NLR levels together with lower albumin concentrations. These findings are highly consistent with existing colorectal surgery literature identifying postoperative inflammatory response as one of the earliest indicators of anastomotic failure. Several studies and reviews have demonstrated that elevated CRP and NLR on postoperative days 3–5 are associated with increased leak risk and may support early diagnostic algorithms [27]. From a clinical perspective, these findings suggest that routinely available laboratory indices may support perioperative risk stratification after CRC surgery. Preoperative HALP may help identify patients with reduced physiological reserve before surgery, whereas postoperative NLR may serve as an early signal of increased complication burden. Importantly, these biomarkers should not replace clinical assessment, surgical judgment, or geriatric evaluation but may complement them in identifying patients who require closer monitoring.

The present study has several limitations. The retrospective, single-center design and the limited number of patients meeting the primary endpoint restricted the statistical power of the study. Multivariable adjustment was not performed because the small number of CCI ≥ 20.9 events relative to the number of clinically relevant covariates would have resulted in a substantial risk of model overfitting and unstable estimates. Moreover, no formal correction for multiple comparisons was applied, increasing the possibility of type I error, and the ROC-derived cutoff values were neither internally nor externally validated. Because the thresholds were derived and evaluated in the same cohort, their diagnostic performance may be optimistic. Therefore, the reported associations should be considered unadjusted and hypothesis-generating, and the proposed cutoff values require validation in larger, prospective, multicenter cohorts, particularly in European populations.

The main contribution of this study lies in the simultaneous evaluation of three readily available inflammatory and immunonutritional indices across multiple perioperative time points in a homogeneous cohort undergoing minimally invasive colorectal cancer surgery. The findings suggest that preoperative and postoperative measurements may provide different types of clinical information: preoperative values may contribute to baseline risk stratification, whereas postoperative changes may be more relevant for surveillance of an evolving adverse course. Nevertheless, the exploratory thresholds identified in this cohort should not be used for clinical decision-making without prospective external validation. Future prospective multicenter studies incorporating comprehensive frailty assessment and larger elderly cohorts are warranted to further define the role of inflammatory biomarkers in modern colorectal surgery [8,24,28,31].

## 5. Conclusions

Perioperative HALP, NLR, and H-index values were associated with clinically relevant postoperative morbidity following minimally invasive colorectal cancer surgery. Among the evaluated postoperative measurements, POD3 NLR demonstrated the highest discriminatory performance; however, it may reflect an already developing postoperative complication rather than provide independent prediction. These findings should be considered exploratory and require confirmation in larger prospective multicenter cohorts with prespecified multivariable analyses and external validation before clinical implementation.

## Figures and Tables

**Table 1:** Baseline clinical, tumor-related, and operative characteristics according to severe postoperative morbidity.

	CCI < 20.9 (n = 81)	CCI ≥ 20.9 (n = 24)	*p*-Value
Age, years	65.0 [57.0–74.0]	68.0 [62.5–72.3]	0.2488
Female sex, n (%)	40 (49.4)	11 (45.8)	0.7610
Body weight, kg	75.0 [66.0–86.0]	80.5 [66.5–85.5]	0.6607
BMI, kg/m^2^	26.0 [23.5–29.1]	26.7 [23.7–28.9]	0.7515
Comorbidities, n (%)	51 (63.0)	18 (75.0)	0.8207
**Surgical approach**			**0.2127**
Laparoscopic	52 (64.2)	12 (50.0)	
Robotic	29 (35.8)	12 (50.0)	
**Type of surgery**			**0.6769**
Right hemicolectomy	30 (37.0)	10 (41.7)	
Left hemicolectomy	2 (2.5)	1 (4.2)	
Sigmoidectomy	3 (3.7)	2 (8.3)	
Anterior resection	39 (48.1)	8 (33.3)	
Abdominoperineal resection (APR)	7 (8.6)	3 (12.5)	
Air-leak test performed, n (%)	35 (43.2)	6 (25.0)	0.1100
Intraoperative complications, n (%)	2 (2.5)	0 (0.0)	0.4392
**Clinical T stage**			**0.0359**
cT1	7 (8.6)	0 (0.0)	
cT2	23 (28.4)	6 (25.0)	
cT3	43 (53.1)	12 (50.0)	
cT4	7 (8.6)	6 (25.0)	
**Histological grade**			**0.6134**
G1–G2	60 (74.1)	19 (79.2)	
G3	21 (25.9)	5 (20.8)	
Neoadjuvant treatment, n (%)	19 (23.5)	6 (25.0)	0.8767
Operative time, min	170.0 [143.8–200.0]	172.5 [145.0–240.0]	0.3714

**Table 2:** Laboratory and immunonutritional parameters associated with severe postoperative morbidity.

Parameter	CCI < 20.9 (n = 81)	CCI ≥ 20.9 (n = 24)	*p*-Value
**Preoperative**			
CRP, mg/L	2.1 [1.0–4.3]	5.5 [2.4–15.6]	**0.0310**
Hemoglobin, g/dL	12.8 [11.5–13.7]	11.2 [10.1–12.3]	**0.0010**
RBC, ×10^12^/L	4.4 [4.2–4.7]	4.2 [4.0–4.5]	**0.0380**
Lymphocyte count, ×10^9^/L	1.5 [1.1–1.8]	1.2 [0.8–1.5]	**0.0101**
H-index	1.4 [1.0–2.5]	2.2 [1.4–4.8]	**0.0148**
HALP score	0.3 [0.2–0.4]	0.2 [0.1–0.2]	**0.0006**
NLR	2.9 [2.3–3.7]	4.4 [2.9–7.1]	**0.0041**
**Postoperative day 1**			
Albumin, g/dL	3.6 [3.3–3.8]	3.4 [3.0–3.5]	**0.0247**
Hemoglobin, g/dL	11.4 [10.0–12.3]	10.1 [9.1–11.1]	**0.0085**
HALP score	0.2 [0.2–0.3]	0.1 [0.1–0.2]	**0.0077**
**Postoperative day 2**			
CRP, mg/L	92.3 [50.9–138.5]	148.7 [92.9–234.8]	**0.0067**
Albumin, g/dL	3.4 [3.3–3.6]	3.2 [2.9–3.3]	**0.0250**
Hemoglobin, g/dL	10.9 [9.9–11.7]	10.1 [8.4–11.3]	**0.0325**
Lymphocyte count, ×10^9^/L	1.3 [1.1–1.9]	0.9 [0.6–1.4]	**0.0301**
H-index	3.8 [1.6–7.1]	6.9 [4.1–11.7]	**0.0329**
HALP score	0.2 [0.1–0.3]	0.1 [0.1–0.2]	**0.0192**
NLR	5.2 [3.4–7.6]	9.0 [6.8–18.2]	**0.0020**
**Postoperative day 3**			
CRP, mg/L	76.6 [45.4–138.0]	142.0 [67.2–263.3]	**0.0235**
Albumin, g/dL	3.3 [3.1–3.6]	3.1 [2.9–3.2]	**0.0048**
Lymphocyte count, ×10^9^/L	1.3 [0.9–1.8]	0.8 [0.5–1.0]	**0.0020**
H-index	1.9 [1.1–3.5]	4.5 [2.3–7.2]	**0.0056**
NLR	3.4 [2.5–5.6]	9.7 [6.0–12.2]	**0.0001**
**Postoperative day 4**			
CRP, mg/L	79.8 [41.6–158.0]	146.0 [94.2–226.0]	**0.0162**
Hemoglobin, g/dL	11.1 [9.9–11.6]	10.1 [9.4–10.5]	**0.0345**

Data are presented as median [IQR]. Only variables demonstrating statistically significant differences between groups are shown. Abbreviations: CCI, Comprehensive Complication Index; CRP, C-reactive protein; HALP, hemoglobin–albumin–lymphocyte–platelet score; NLR, neutrophil-to-lymphocyte ratio; POD, postoperative day; RBC, red blood cells. *p*-values were calculated using the Mann–Whitney U test.

**Table 3:** Diagnostic performance of selected laboratory-derived indices for predicting postoperative complications.

Variable	Sensitivity (%)	Specificity (%)	Cutoff	AUC (95% CI)	*p*-Value
**Preoperative period**					
H-index	80.0	51.72	>1.41	0.68 (0.57–0.78)	**0.0081**
HALP	76.5	87.70	≤0.19	0.78 (0.66–0.87)	**0.0003**
NLR	55.0	82.76	>3.83	0.72 (0.60–0.81)	**0.0022**
**Postoperative day 1**					
H-index	40.0	72.22	>9.89	0.54 (0.42–0.66)	0.0785
HALP	73.3	86.11	≤0.15	0.74 (0.60–0.85)	**0.0074**
NLR	35.0	88.89	>12.36	0.61 (0.49–0.72)	0.1480
**Postoperative day 2**					
H-index	76.47	55.56	>3.86	0.69 (0.54–0.82)	**0.0182**
HALP	86.67	63.16	≤0.21	0.74 (0.56–0.87)	**0.0082**
NLR	88.24	62.96	>5.70	0.78 (0.63–0.89)	**0.0001**
**Postoperative day 3**					
H-index	100.0	47.37	>1.66	0.77 (0.63–0.88)	**0.0003**
HALP	93.75	42.55	≤0.16	0.61 (0.48–0.73)	0.0699
NLR	91.67	73.68	>5.23	0.87 (0.74–0.95)	**<0.0001**

## Data Availability

The original contributions presented in this study are included in the article. Further inquiries can be directed to the corresponding author.

## Abbreviations

ASAR	Abdominoperineal Anterior Resection
AUC	Area Under the Receiver Operating Characteristic Curve
BMI	Body Mass Index
CCI	Comprehensive Complication Index
CGA	Comprehensive Geriatric Assessment
CI	Confidence Interval
CRC	Colorectal Cancer
CRP	C-Reactive Protein
GNRI	Geriatric Nutritional Risk Index
HALP	Hemoglobin–Albumin–Lymphocyte–Platelet Score
H-index	Host Index
ICU	Intensive Care Unit
IQR	Interquartile Range
IRB	Institutional Review Board
LOS	Length of Hospital Stay
NLR	Neutrophil-to-Lymphocyte Ratio
PCT	Procalcitonin
PNI	Prognostic Nutritional Index
POD	Postoperative Day
RBC	Red Blood Cell Count
ROC	Receiver Operating Characteristic
TO	Textbook Outcome
TOO	Textbook Oncological Outcome
TNM	Tumor-Node-Metastasis Classification
